# Advancing *Mytilus galloprovincialis* Primary Cell Cultures as Models for Bioactive Compound Screening and Environmental Safety Assessment

**DOI:** 10.3390/biology15141111

**Published:** 2026-07-09

**Authors:** Yanwen Ma, Lucia De Marchi, Gianfranca Monni, Joanna Giannessi, Valentina Meucci, Alessio Lenzi, Marzia Vasarri, Donatella Degl’Innocenti, Carlo Pretti

**Affiliations:** 1 Department of Veterinary Sciences, University of Pisa, Viale delle Piagge 2, 56124 Pisa, Italy; y.ma8@studenti.unipi.it (Y.M.); gianfranca.monni@unipi.it (G.M.); valentina.meucci@unipi.it (V.M.); alessio.lenzi@unipi.it (A.L.); or pretti@cibm.it (C.P.); 2 Interuniversity Center of Marine Biology and Applied Ecology “G. Bacci” (CIBM), Viale N. Sauro 4, 57128 Livorno, Italy; joanna.giannessi@phd.unipi.it; 3 Department of Experimental and Clinical Biomedical Sciences, University of Florence, Viale Morgagni 50, 50134 Florence, Italy; marzia.vasarri@unifi.it (M.V.); donatella.deglinnocenti@unifi.it (D.D.)

**Keywords:** *Mytilus galloprovincialis*, primary bivalve cell culture, bioindicators, in vitro ecotoxicology, seeding density, digestive gland, gills, metabolic activity

## Abstract

Marine bivalves represent promising experimental models for the development of in vitro approaches in environmental biology, ecotoxicology, and bioactive compound screening. However, their application remains limited compared with that of more established vertebrate and mammalian cell systems, mainly due to the lack of standardized and reproducible protocols for maintaining primary cell cultures. This study aimed to establish and optimize a short-term primary cell culture protocol using gill and digestive gland tissues from the marine mussel *Mytilus galloprovincialis*. Primary cultures were prepared at four initial cell densities, ranging from 2.5 to 10 × 10^6^ cells mL^−1^, and monitored over 72 h through cell density evaluation and metabolic activity assessment. Cells from both tissues were successfully maintained in short-term culture, predominantly showed rounded to spheroidal morphologies. Nevertheless, tissue-specific responses were observed. Gill-derived cultures displayed greater morphological homogeneity and stability across the tested densities, whereas digestive gland cultures showed greater cellular heterogeneity, aggregation, and stronger density-dependent responses. Metabolic activity remained stable during the first 24 h, while more evident differences among cell densities emerged after 48 and 72 h. Overall, intermediate cell densities, particularly 5 × 10^6^ and 7.5 × 10^6^ cells mL^−1^, provided the most suitable balance between cell maintenance and metabolic performance. This optimized methodological framework supports the further development of mussel primary cell cultures as reproducible and ecologically relevant in vitro models for studying early cellular responses and for preliminary environmental safety assessment.

## Simple Summary

Marine bivalves are increasingly considered promising experimental models for in vitro approaches, although their use remains limited compared with that of other established cell systems. Primary cell cultures from mussels may provide useful tools for investigating early cellular responses under controlled laboratory conditions and for supporting the preliminary evaluation of bioactive compounds or environmental stressors. In this study, short-term primary cultures were developed from the gills and digestive gland of *Mytilus galloprovincialis* and maintained for 72 h at different initial cell densities. Gill-derived cultures showed greater homogeneity and stability, while digestive gland cultures were more variable and density-dependent. Intermediate densities, especially 5 × 10^6^ and 7.5 × 10^6^ cells mL^−1^, provided the most suitable conditions. This protocol supports the development of reproducible mussel-based in vitro models for experimental biology, ecotoxicology, and environmental safety assessment.

## 1. Introduction

Marine bivalves, including marine mussels, are widely used as sentinel organisms in ecotoxicology because of their filter-feeding lifestyle, their continuous interaction with the surrounding water column, and their capacity to accumulate contaminants from the environment [1,2]. Consequently, mussel-based experimental systems are highly valuable for investigating pollutant effects, tissue-specific stress responses, and host–environment interactions under controlled conditions [3].

In this context, primary cell cultures represent an attractive complementary approach to whole-animal assays, as they provide reductionist in vitro platforms while still retaining part of the physiological identity of the source tissue [4,5]. Recent advances in marine invertebrate and molluscan cell culture have highlighted both the potential and the current limitations of these systems, emphasizing the need for optimized, tissue-specific protocols and better-characterized primary cultures [5,6,7]. In bivalves, primary cultures derived from tissues such as gills, the digestive gland, mantle, or hemocytes are increasingly recognized as useful tools for investigating cellular responses related to ecotoxicology, immunotoxicity, oxidative stress, and pollutant exposure [6,7,8,9]. Beyond conventional pollutant assessment, reliable bivalve in vitro models are also valuable for the early-stage screening of bioactive compounds, including marine natural products and other xenobiotics intended for biomedical, antifouling, aquaculture, cosmetic, or broader aquatic applications [8,9,10,11]. This is particularly relevant in scenarios where bivalves and fouling-associated organisms may represent ecologically meaningful target species or organisms directly exposed to these compounds [8,9]. In this regard, cellular models derived from bivalve tissues can provide valuable preliminary insights into potential cytotoxic, immunological, oxidative, or metabolic effects prior to subsequent whole-organism testing [5,6,7,8,9].

Despite this potential, the establishment of stable primary cultures from marine bivalves remains technically demanding. Compared with mammalian cell systems, bivalve primary cells are often characterized by marked heterogeneity, limited survival in vitro, variable attachment behavior, and a strong dependence on species-specific osmotic and nutritional conditions [12,13]. These limitations have restricted the widespread standardization of mussel cell culture protocols and, consequently, their broader application in ecotoxicological and methodological studies, and in the context of emerging xenobiotics with dual relevance for pharmacological development and environmental safety assessment. Early efforts demonstrated that short-term cultures could be obtained from mussel tissues but also highlighted progressive declines in cell integrity and survival over time, with clear differences among cell types and tissues [6].

An additional unresolved issue concerns the influence of initial seeding density on the performance of short-term mussel primary cultures. In conventional cell culture systems, seeding density is a critical experimental variable because it affects attachment, survival, proliferation, cell communication, nutrient consumption, waste accumulation, metabolic activity, and the reproducibility of viability-based assays [14,15]. This is particularly relevant when metabolic endpoints are used, as assay signals may reflect not only viable cell number but also density-dependent changes in mitochondrial activity and cellular metabolic state [16]. In marine invertebrate and bivalve primary cultures; however, seeding density has rarely been evaluated as an independent optimization parameter. This represents an important gap, since reliable marine invertebrate cell culture remains constrained by species-, tissue- and cell-type-specific requirements, including medium composition, osmolarity, cell selection and maintenance conditions [5,7,13]. Both excessive and insufficient seeding densities may compromise culture performance. High densities can promote crowding, nutrient and oxygen depletion, accumulation of metabolic by-products and altered metabolic output, whereas low densities may reduce contact-dependent support, impair attachment, and increase cell loss during short-term incubation [15,17].

A further methodological consideration concerns the interpretation of metabolic viability assays, whose readouts do not exclusively reflect cell number [7]. Resazurin- and tetrazolium-based assays are widely employed as indirect proxies of cell viability and proliferative activity; however, their signals are subject to confounding influences, including the physiological state of the cells, per-cell metabolic efficiency, and density-dependent regulation of metabolic activity [18,19,20,21,22]. Consequently, assessment of short-term culture performance based solely on such readouts may yield an incomplete picture unless complemented by direct cell enumeration. In this context, the relationship between cell number and metabolic output in short-term mussel primary cultures is likely to be non-linear and may vary as a function of tissue origin, seeding density, and culture duration.

Nevertheless, such limitations do not preclude the applicability of mussel primary cultures as experimental models, provided that experimental conditions are designed in accordance with their inherent biological properties, short-term viability, and methodological boundaries. Rather than being conceived as long-term proliferative systems, these cultures are more appropriately framed as short-term, tissue-derived cellular models designed to assess early functional responses under controlled in vitro conditions. This makes them particularly suitable for mechanistic studies in which the objective is to assess rapid cellular perturbations, compare tissue-specific responsiveness, or optimize methodological parameters affecting culture performance. From an ecotoxicological perspective, this is particularly relevant because many contaminant-induced effects are initiated at the cellular level before they become detectable as tissue-level damage, organismal impairment, or population-level consequences [7,23].

Among the tissues available for mussel cell culture, the digestive gland has traditionally received the greatest attention, largely due to its central role in digestion, assimilation, and xenobiotic biotransformation, which makes it a key target for toxicological investigations [24]. However, gills are equally relevant from biological and ecotoxicological perspectives. As the primary interface with the external aquatic environment, they are continuously exposed to suspended particles, dissolved contaminants, and microorganisms, and play a fundamental role in filtration and environmental sensing [25]. These functional differences are also reflected at the cellular level, as both tissues are deeply involved in the regulation of oxidative stress and immune or inflammation-like responses. The digestive gland is a major site of xenobiotic metabolism associated with reactive oxygen species (ROS) production and antioxidant defense activation, whereas gills act as a first-line barrier where early oxidative and immune-related responses to waterborne contaminants are initiated [26,27,28]. These pathways are particularly relevant in the context of bioactive compounds targeting pathways such as oxidative stress and inflammation in biomedical research.

In this context, methodological improvements led to the development of soft dissociation procedures for the isolation of cells from the gills and digestive gland of *Mytilus edulis*, providing an important basis for short-term primary culture approaches in mussels [29]. Since then, additional studies and reviews on marine invertebrate and molluscan cell culture have further highlighted the need to optimize tissue-specific protocols, improve inoculum preparation, define suitable culture conditions, and better characterize cell identity and functionality in vitro [5,6,7,30]. Systematic advances in primary cultures from adult oyster tissues have demonstrated the potential of bivalve-derived cultures to maintain metabolically active cells over extended short-term periods, supporting their use in ecotoxicology, immunology, virology, and disease-related studies [5,30]. More recently, short-term in vitro assays using bivalve hemocytes, including cells from *Mytilus galloprovincialis*, have further confirmed the relevance of molluscan cellular models for assessing immune-related, functional, and toxicological responses to environmental contaminants and bioactive compounds [8,31]. However, these approaches were not specifically designed to define optimal culture conditions for standardized and reproducible in vitro testing using gill and digestive gland cells. Rather, early mussel protocols mainly addressed cell isolation efficiency, short-term viability, and basic functional endpoints after dissociation [29]. Consequently, important methodological variables, such as the most appropriate initial seeding density, the comparative behavior of different source tissues, the maintenance of cell abundance over time, and the relationship between morphology and metabolic performance, remain insufficiently explored.

Taking this evidence into account, the present study aimed to refine and extend the methodological framework for short-term primary cultures of *M. galloprovincialis* by adapting the soft dissociation approach originally described for *M. edulis* by Faucet et al. [29]. This protocol was selected as the methodological starting point because it specifically describes the isolation of both gill and digestive gland cells from a *Mytilus* species, making it more directly comparable to the present study than more recent works focused on other molluscan species, tissues, or cell types. Thus, our aim was not simply to reproduce an earlier protocol, but to adapt and further optimize a tissue-relevant approach for short-term primary cultures in *M. galloprovincialis.* While the reference protocol established cultures at 4 × 10^6^ cells mL^−1^, the present study expanded upon this condition by testing four initial seeding densities: 10 × 10^6^, 7.5 × 10^6^, 5 × 10^6^, and 2.5 × 10^6^ cells mL^−1^. Cell number and viability were monitored after 24, 48, and 72 h of culture, allowing the temporal performance of each density condition to be assessed. This approach provides additional methodological information on density-dependent cell maintenance and metabolic performance, contributing to the standardization of mussel-based in vitro models for environmental and ecotoxicological applications.

## 2. Materials and Methods

### 2.1. Chemicals and Reagents Preparation

All chemicals and reagents used for primary mussel cell isolation and culture were of analytical grade. The reagents included sodium chloride (NaCl), potassium chloride (KCl), ethylenediaminetetraacetic acid (EDTA), calcium chloride dihydrate (CaCl_2_·2H_2_O), magnesium sulphate (MgSO_4_), magnesium chloride hexahydrate (MgCl_2_·6H_2_O), HEPES buffer, and Leibovitz’s L-15 culture medium. In addition, penicillin G, streptomycin sulphate, gentamycin sulphate, and fetal bovine serum (FBS) were used for medium supplementation and contamination control. Unless otherwise specified, all reagents were purchased from Sigma-Aldrich© (Milan, Italy).

All reagents were prepared following the formulation reported by Faucet et al. [29].

The modified Leibovitz’s L-15 medium used for primary culture was prepared to an osmolarity of 1100 mOsm and adjusted to pH 7.3. Its composition was as follows: L-15 medium, 15.08 g L^−1^; NaCl, 20.2 g L^−1^; KCl, 0.54 g L^−1^; CaCl_2_, 0.6 g L^−1^; MgSO_4_, 1.0 g L^−1^; and MgCl_2_, 3.9 g L^−1^. Distilled water was added to volume, and the pH was adjusted to 7.3. The medium was sterilized by filtration through a 0.22 µm membrane filter. Immediately before use, the medium was supplemented with 10% FBS (*v*/*v*) and 1% gentamycin sulphate at 1 mg mL^−1^.

The calcium- and magnesium-free saline buffer (CMFS) used for tissue washing and dissociation was also prepared according to the reference work, with a final osmolarity of 1100 mOsm and pH 7.3. The formulation consisted of HEPES, 5.2 g L^−1^; NaCl, 29.22 g L^−1^; KCl, 0.93 g L^−1^; and EDTA, 1.86 g L^−1^. Distilled water was added to volume, the pH was adjusted to 7.3, and the solution was sterilized by filtration through a 0.22 µm membrane. Immediately before use, CMFS was supplemented with 1% gentamycin sulphate to obtain a final concentration of 1 mg mL^−1^. A gentamycin stock solution was prepared at 10 mg mL^−1^ in either CMFS buffer or modified L-15 medium, sterilized through a 0.22 µm filter, aliquoted, and stored at −20 °C until use.

For the analyses, cell viability was assessed using the alamarBlue™ Cell Viability Reagent (Thermo Fisher Scientific, Waltham, MA, USA), a resazurin-based, ready-to-use solution that enables the quantitative measurement of metabolic activity in living cells. This assay is based on the reduction in resazurin to the fluorescent compound resorufin by metabolically active cells, providing a sensitive and non-destructive indicator of cell viability [32]. The reagent was used according to the manufacturer’s instructions for microplate-based assays, as previously described for viability assessment in cell culture systems.

### 2.2. Sampling and Preparation of the Organisms

Specimens of *M. galloprovincialis* (approximately 4.0 ± 0.5 cm shell length) were obtained from long-line mussel farming facilities located in the Gulf of La Spezia (44°06′ N, 9°50′ E; Ligurian Sea, north-western Mediterranean Sea, Italy), operated by the *Mitilicoltori Spezzini* producers’ organization. This area is characterized by semi-enclosed coastal conditions and is widely used for commercial mussel aquaculture. Collected specimens were transported to the Laboratory of Pharmacology and Toxicology, Department of Veterinary Sciences, University of Pisa (Pisa, Italy). In the laboratory, mussels were acclimated in filtered and continuously aerated seawater under controlled conditions (temperature: 16–18 °C; salinity: 38–40; dissolved oxygen: >90% saturation; pH: 8.0–8.2) until use in the experiments. The day after acclimation, mussels were first cleaned of epibiotic growth, and the shell surface was decontaminated with 75% ethanol and allowed to dry. To reduce microbial contamination and improve cell quality before culture initiation, the animals were maintained in aquaria at a density of 10 animals per 3 L^−1^ of seawater. In the original method, one pool was processed immediately after overnight incubation in seawater containing antibiotics, whereas additional pools were held for two days in clean, oxygenated seawater, which was replaced daily, followed by overnight antibiotic exposure. In the present work, the 48 h pre-conditioning step in clean oxygenated seawater was retained prior to tissue collection because this condition was associated in the reference study with improved cell quality and reduced contamination risk. The water was changed every 24 h [29]. Before antibiotic pre-treatment, the organisms were divided into four glass beakers containing sterile seawater at a density of 5 animals L^−1^. Penicillin G and streptomycin were added to the seawater at final concentrations of 0.06 mg mL^−1^ and 0.1 mg mL^−1^, respectively. Mussels were maintained under this antibiotic pre-treatment overnight, for approximately 16 h, at the same controlled temperature used during acclimation, namely 16–18 °C. This step was performed to reduce the risk of microbial contamination and thereby improve aseptic conditions during subsequent dissection and tissue washing procedures.

### 2.3. Dissection and Tissue Washing

All dissections and manipulations were performed under sterile conditions in a laminar flow hood in a thermoregulated room maintained at 18 ± 1 °C. A pool of 20 mussels was opened aseptically with a scalpel, and both digestive glands and gills were collected using sterile scissors and forceps. Both tissues were selected because of their complementary physiological roles in uptake, filtration, xenobiotic metabolism, and cellular responses associated with oxidative stress and immune/inflammation-like pathways. Tissues were carefully trimmed to remove traces of mantle and kidney; in the case of digestive glands, the crystalline style was also removed, following the original procedure. Tissue samples were then placed in pre-weighed sterile Petri dishes on ice and weighed before processing. Each tissue type was transferred into sterile beakers and washed twice in CMFS buffer containing 1% gentamycin sulphate. The volume used in the reference method was 10 mL of CMFS buffer per digestive gland or per pair of gills. Washing was performed under gentle stirring for 5 min. After each wash, the washing solution was filtered through a sterile inox strainer, and the tissue was recovered for the subsequent wash or dissociation step. This repeated washing step was essential to reduce mucus, debris, and microbial contamination before cell release.

### 2.4. Soft Dissociation

#### 2.4.1. Digestive Gland

Digestive glands were placed in a sterile beaker containing 50 mL of CMFS buffer supplemented with 1% gentamycin and minced into small pieces of approximately 2 mm using sterile scissors. The fragmented tissue was then transferred to a sterile beaker containing 250 mL of fresh CMFS buffer supplemented with 1% gentamycin and subjected to gentle magnetic stirring at 100 rpm for 2 h. During this dissociation period, aliquots of the cell suspension were removed every 30 min and replaced with fresh CMFS buffer containing 1% gentamycin, thereby maintaining dissociation efficiency while limiting prolonged exposure of released cells to tissue debris. The resulting suspension was filtered successively through sterile inox strainers, 100 µm nylon mesh, and 37 µm nylon mesh to remove undigested material and large aggregates. The filtrate was then centrifuged first at 200× *g* for 10 min to recover smaller cell fractions. Pellets from both centrifugation steps were resuspended in modified L-15 culture medium and centrifuged again at 200× *g* for 10 min, to remove residual CMFS buffer before final suspension in culture medium [29]. Pelleted cells were resuspended in modified L-15 culture medium supplemented with 10% FBS and 1% gentamycin, and cell density was measured.

#### 2.4.2. Gills

Gills were placed in a sterile beaker containing 50 mL CMFS buffer supplemented with 1% gentamycin and finely excised and chopped using sterile scissors. The chopped gill tissue was then transferred into a sterile beaker containing 250 mL fresh CMFS buffer with 1% gentamycin and gently stirred at 100 rpm for 1 h. Compared with digestive gland tissue, gills required a shorter dissociation time because of their more fragile structure and easier release of cells into suspension. Following dissociation, aliquots of the cell suspension were filtered through sterile inox strainers, followed by 100 µm and 37 µm nylon meshes. The filtrate was centrifuged at 200× *g* for 10 min to pellet the cells. The resulting pellet was resuspended in modified L-15 culture medium and centrifuged again at 200× *g* for 10 min to eliminate residual CMFS buffer. The final pellet was then resuspended in culture medium for counting and plating. Pelleted cells were resuspended in culture modified L-15 medium supplemented with 10% FBS and 1% gentamycin and cell density was measured.

### 2.5. Cell Counting, Seeding Densities and Culture Conditions

After isolation, both digestive gland- and gill-derived cell suspensions were homogenized by repeated slow pipetting to obtain a uniform cell distribution and to minimize cell aggregation. Cell concentration was then determined by manual counting using a Bürker counting chamber under an inverted microscope (Leica Mateo TL; Leica Microsystems, Wetzlar, Germany). Briefly, a representative aliquot of each cell suspension was loaded into the haemocytometer chamber, and cells were counted in selected grid squares. When cell density was too high for reliable enumeration, appropriate dilutions were prepared in modified L-15 medium before counting. Cell concentration was calculated according to the following formula:

$$Cell\;{mL}^{-1}=mean\;number\;of\;cells\;counted\;per\;square\times 10^{4}\times dilution\;factor$$

The calculated cell concentrations were then used to adjust the suspensions to the four experimental seeding densities: 10 × 10^6^, 7.5 × 10^6^, 5 × 10^6^, and 2.5 × 10^6^ cells mL^−1^. For each tissue type and density, aliquots of the adjusted cell suspensions were seeded into 96-well plates containing modified L-15 medium supplemented with 10% FBS and 1% gentamicin, in a final volume of 100 μL per well. Cultures were maintained at 18 °C under static conditions. For each cell density and sampling time, six independent replicate wells (n = 6) were analyzed at the beginning of the experiment (T0) and after 24 h (T24), 48 h (T48), and 72 h (T72) of culture. Dedicated replicate wells were assigned to each sampling time point and were never reused at later time points, ensuring that the culture integrity of the remaining wells was fully preserved throughout the experiment. At each time point, a 10 µL aliquot was then withdrawn for direct cell enumeration in the Bürker counting chamber prior to the addition of the alamarBlue reagent to the remaining volume. This sequential approach ensured that cell count data and metabolic activity measurements were obtained from the same well at the same time point, without mutual interference. A cell-free medium control was included as a blank to correct for background fluorescence/absorbance potentially generated by the culture medium, supplements, or assay reagents in the absence of cells. A positive control was also included to validate the responsiveness of the viability assay, consisting of cells exposed to a cytotoxic condition (10% DMSO), which induced a marked reduction in cell viability.

### 2.6. Culture Cells Density and Viability

#### 2.6.1. Microscopic Monitoring of Cell Number

Representative images at 10× and 20× magnification were acquired for each experimental time point and initial cell density to document the main morphological features of the cultures. These images were used to assess cell shape, spatial distribution, degree of aggregation, adherence patterns, and possible density-dependent changes over time. Image acquisition also allowed the visual monitoring of culture conditions throughout the incubation period, including changes in cell organization, maintenance of cellular integrity, and the presence of cellular debris or signs of culture deterioration.

#### 2.6.2. Cell Viability

Cells were seeded in microplates in the appropriate culture medium and allowed to stabilize under the selected experimental conditions. The alamarBlue reagent was then added directly to each well according to the manufacturer’s recommended volume for microplate-based assays. For 96-well plates, 10 µL of reagent was added to 90 µL of cell suspension, corresponding to a 10% final assay volume. After reagent addition, plates were incubated for 1–4 h to allow intracellular reduction in resazurin. Fluorescence was subsequently measured using a microplate reader (BioTek Sinergy HTX; BioTek Instruments, Winooski, VT, USA) with excitation and emission wavelengths set at 560 and 590 nm, respectively. Background fluorescence was corrected by including control wells containing culture medium and alamarBlue reagent but no cells. Fluorescence detection was selected due to its higher sensitivity compared to absorbance-based measurements, enabling more accurate detection of variations in metabolic activity. The fluorescence signal was interpreted as proportional to the metabolic competence of viable cells, whereas reduced fluorescence indicated decreased viability and/or impaired cellular metabolism (Thermo Fisher Scientific protocol).

### 2.7. Data Analyses

#### 2.7.1. Data Processing and Fold-Change Calculation

Fluorescence data obtained from the alamarBlue assay were processed separately for digestive gland- and gill-derived primary cultures. For each tissue type, time point and initial seeding density, raw fluorescence values were first corrected for background signal by subtracting the mean fluorescence value of the corresponding cell-free culture medium. Corrected fluorescence values were then used to calculate the mean and standard deviation for each experimental condition. To evaluate the temporal evolution of metabolic activity, data were expressed as fold changes relative to the corresponding T0 value. This calculation was performed independently for each tissue type and for each initial seeding density. The mean corrected fluorescence measured at T0 was used as the baseline reference and was set to 1.00. Fold change values at subsequent time points were calculated according to the following equation:

$$Fold\;change\;vs.\;T0=\frac{{Mean\;corrected\;fluorescence\;at\;time}_{x}}{Mean\;corrected\;fluorescence\;at\;T0}$$

where time_x_ corresponded to 24, 48 or 72 h of culture. This approach allowed the temporal increase or decrease in metabolic activity to be evaluated independently of the initial fluorescence signal associated with each seeding density. Fold-change calculations were performed separately for digestive gland and gill cultures seeded at 10 × 10^6^, 7.5 × 10^6^, 5 × 10^6^ and 2.5 × 10^6^ cells mL^−1^.

#### 2.7.2. Relative Metabolic Activity (RMA)

For each experimental time point, Fluorescence Units (RFU) values were measured for each initial seeding density and averaged across replicate wells after background subtraction. Mean RFU values were used as indirect indicators of cellular metabolic activity, as the alamarBlue assay relies on the reduction in resazurin to resorufin by metabolically active viable cells [22,33,34]. To compare metabolic responses among seeding densities, data were normalized within each time point. RMA was calculated by expressing the mean background-corrected RFU of each density condition as a percentage of the highest mean RFU recorded at the same time point. This approach allowed density-dependent differences in metabolic performance to be compared independently of variations in absolute signal intensity between time points, following standard recommendations for metabolic viability assays [35]. For each time point, the highest mean RFU value observed among all cell densities was defined as the reference value (RFU_max_). RMA was then calculated according to the following equation:

$$RMA\left(\%\right)=\frac{{RFU}_{t}}{{RFU}_{max}}\times 100$$

where RFU_t_ represents the mean RFU measured for a given cell density at a specific time point, and RFU_max_ corresponds to the highest mean RFU value observed among all conditions at that same time point. The condition showing the highest metabolic signal was set to 100%, and all other conditions were expressed as a percentage relative to this value. In cases where background subtraction resulted in negative RFU values, the corresponding RMA values were interpreted as signals below the assay detection threshold rather than biologically meaningful negative metabolic activity. Such values were therefore considered indicative of signals lower than the background level of the assay.

#### 2.7.3. Relationship Between Metabolic Activity and Cell Density

To investigate whether metabolic activity reflected the actual number of cells present under each experimental condition, the percentage of RMA was mathematically compared with normalized cell density values. Total cell counts obtained for each treatment were first normalized within each time point as a percentage of the maximum observed cell density according to the following equation:

$$Cell\;density\;relative\;\left(\%\right)=\frac{{Tot}_{i}}{{Tot}_{max}}\times 100$$

where Tot_i_ represents the total number of cells measured for a given condition and Tot_max_ corresponds to the highest cell density observed at the same time point. These normalized values were then compared with the RMA (%) previously calculated. To quantitatively assess the relationship between metabolic activity and cell abundance, a metabolic efficiency index was calculated as the ratio between RMA and normalized cell density. Values close to 1 indicate that metabolic activity is proportional to cell abundance, whereas values above or below 1 indicate, respectively, higher or lower metabolic performance than would be expected based solely on cell number.

### 2.8. Statistical Analyses

Corrected alamarBlue fluorescence was used as the primary endpoint for inferential statistical analysis. Raw fluorescence readings obtained from gill- and digestive gland-derived primary cultures were corrected for background signal by subtracting the mean fluorescence of the corresponding cell-free medium. Primary cultures from both tissues were established at four initial seeding densities of 10 × 10^6^, 7.5 × 10^6^, 5 × 10^6^ and 2.5 × 10^6^ cells mL^−1^, and monitored at 0, 24, 48 and 72 h. Before performing the parametric tests, data distribution and homogeneity of variance were assessed. Normality was evaluated using the Shapiro–Wilk test, while homogeneity of variance among groups was assessed using Levene’s test. After confirming that the assumptions for parametric analysis were satisfied, corrected fluorescence data were analyzed separately for each tissue using two-way ANOVA, with experimental time point and initial seeding density as fixed factors. Post hoc comparisons were performed using Tukey’s multiple comparisons test. Statistical significance was accepted at *p* < 0.05. Statistical analyses and graphical outputs were generated using GraphPad Prism^®^ version 8.4.3 (GraphPad Software, Boston, MA, USA). Data are presented as box-and-whisker plots in which the central line represents the median, the box spans the interquartile range (IQR, 25th–75th percentile), and the whiskers extend to the minimum and maximum observed values. All individual replicate data points (n = 6) are superimposed on the plots. The statistical annotations shown in the figures refer only to comparisons among initial seeding densities within the same experimental time point. Comparisons across different times were included in the statistical analysis but are not indicated in the figures to improve readability.

Normalized and derived parameters, including RMA, relative cell density, metabolic efficiency index and Δ, were used as descriptive indices. These parameters were not included in inferential statistical analyses unless calculated at the replicate level.

## 3. Results

### 3.1. Gill-Derived Primary Cultures

#### 3.1.1. Time- and Density-Dependent Morphology

**Figure 1:**
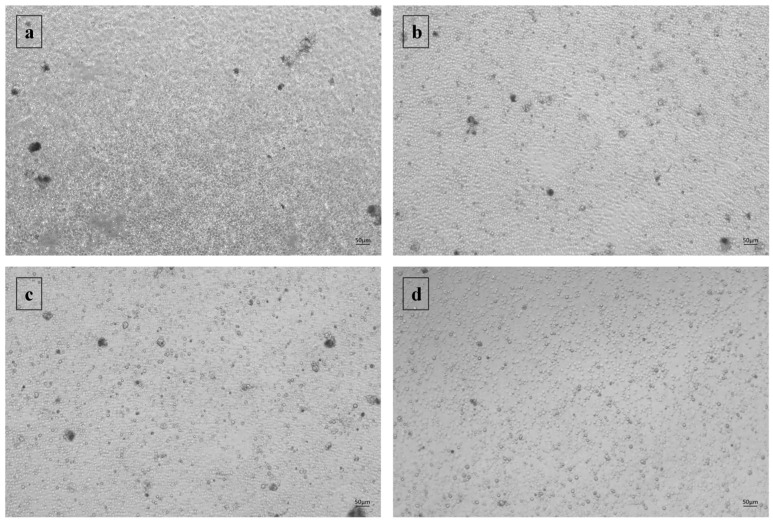
Representative light microscopy images (10×) of *M. galloprovincialis* gill-derived cell suspensions immediately after seeding (T0) at different initial cell densities. Panels (**a**–**d**) correspond to 10 × 10^6^, 7.5 × 10^6^, 5 × 10^6^ and 2.5 × 10^6^ cells mL^−1^, respectively.

**Figure 2:**
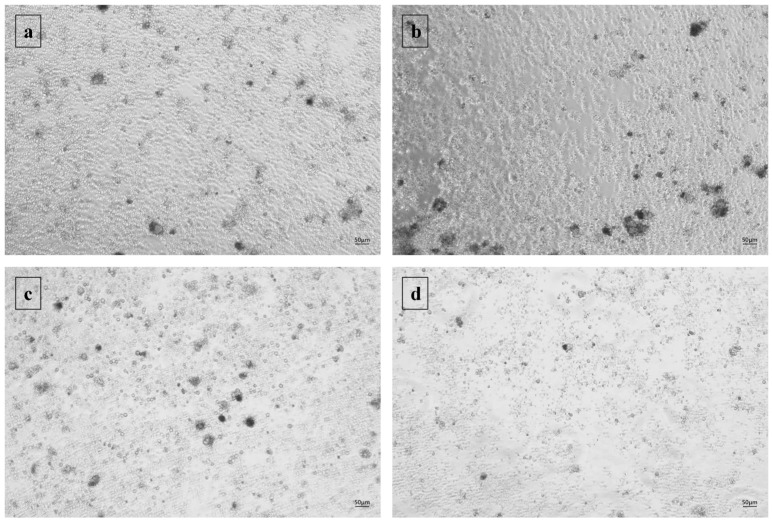
Representative light microscopy images (10×) of *M. galloprovincialis* gill-derived cell suspensions immediately after 24 h of incubation (T24) at different initial cell densities. Panels (**a**–**d**) correspond to 10 × 10^6^, 7.5 × 10^6^, 5 × 10^6^ and 2.5 × 10^6^ cells mL^−1^, respectively.

**Figure 3:**
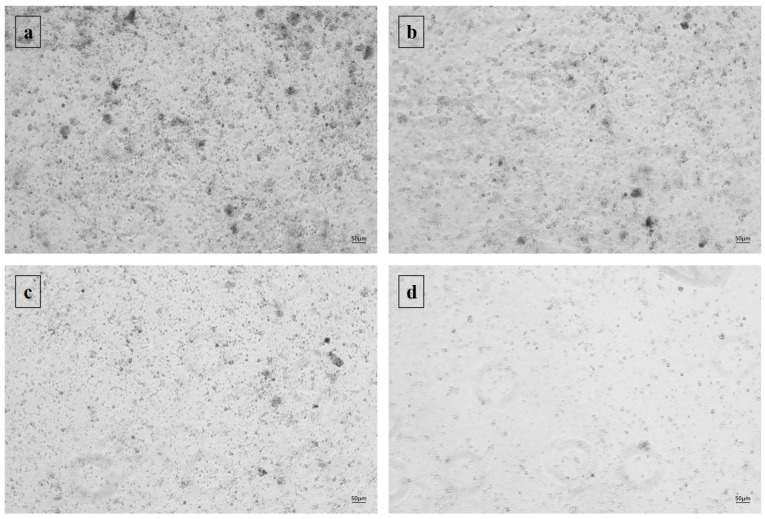
Representative light microscopy images (10×) of *M. galloprovincialis* gill-derived cell suspensions immediately after 48 h of incubation (T48) at different initial cell densities. Panels (**a**–**d**) correspond to 10 × 10^6^, 7.5 × 10^6^, 5 × 10^6^ and 2.5 × 10^6^ cells mL^−1^, respectively.

**Figure 4:**
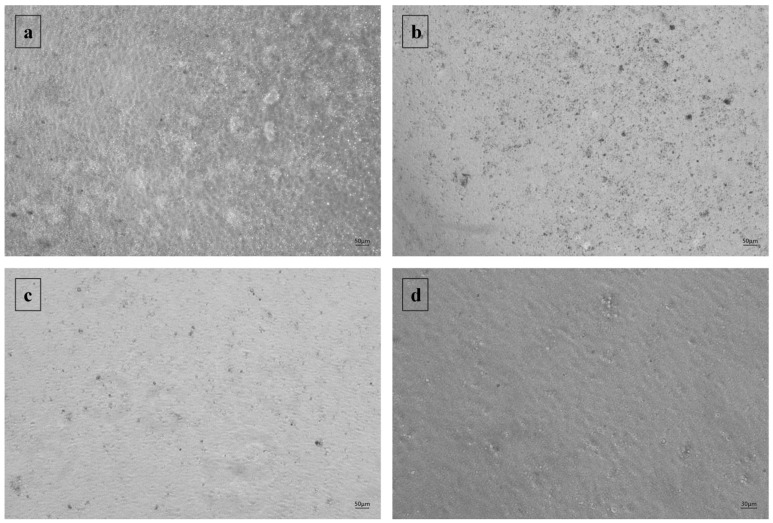
Representative light microscopy images (10×) of *M. galloprovincialis* gill-derived cell suspensions immediately after 72 h of incubation (T72) at different initial cell densities. Panels (**a**–**d**) correspond to 10 × 10^6^, 7.5 × 10^6^, 5 × 10^6^ and 2.5 × 10^6^ cells mL^−1^, respectively.

**Figure 5:**
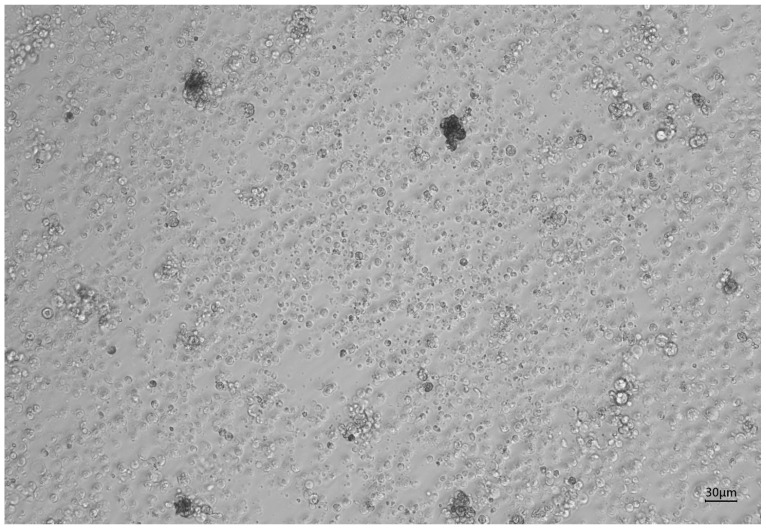
Detailed microscopic view of *M. galloprovincialis* gill-derived cell suspensions at 20× magnification after 48 h of incubation at a cell density of 10 × 10^6^ cells mL^−1^.

Figure 1, Figure 2, Figure 3 and Figure 4 show the microscopic appearance of mussel-derived primary cell cultures at different incubation times: T0, T24, T48 and T72, respectively. Within each figure, panels a–d correspond to the tested initial seeding densities of 10 × 10^6^, 7.5 × 10^6^, 5 × 10^6^ and 2.5 × 10^6^ cells mL^−1^, respectively. Microscopic assessment showed that mussel-derived cells maintained a rounded to spheroidal morphology across all tested seeding densities and incubation times. Individual cells were consistently smaller than the 30 µm reference scale and were characterized by a refractile, translucent cytoplasm, well-defined cell margins and preserved cellular outlines, suggesting the maintenance of gross morphological integrity under the tested culture conditions (Figure 5).

#### 3.1.2. Fluorescence Intensity

**Figure 6:**
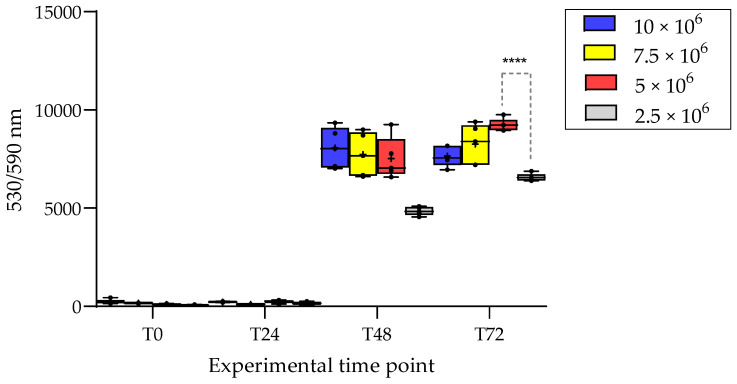
Time-dependent changes in fluorescence intensity in gill-derived primary cultures. **** *p* < 0.0001.

No significant differences in fluorescence intensity were observed between T0 and T24, indicating that metabolic activity remained statistically unchanged during the first 24 h of culture. In contrast, fluorescence values recorded at T48 and T72 were significantly higher than those measured at T0 and T24 across all tested seeding densities (*p* < 0.0001), suggesting an overall increase in metabolic activity after 48 h of culture. At T48, fluorescence values ranged from 6454 to 8065 among the different seeding densities, with no significant differences detected between them. Similarly, at T72, fluorescence intensity did not differ significantly among most seeding densities. The only significant difference observed within this time point was between 5 × 10^6^ and 2.5 × 10^6^ cells mL^−1^, with higher fluorescence in the 5 × 10^6^ cells mL^−1^ condition (mean difference = 2670; 95% CI: 969.5–4370; *p* < 0.0001; Figure 6). When comparing T48 and T72, fluorescence intensity increased significantly only in cultures seeded at 5 × 10^6^ cells mL^−1^ (mean difference = 1716; 95% CI: 15.32–3416; *p* = 0.0458). In addition, T72 values recorded at 7.5 × 10^6^ and 5 × 10^6^ cells mL^−1^ were higher than the T48 values observed at 2.5 × 10^6^ cells mL^−1^ (Figure 5).

In gill-derived cultures, the corrected fluorescence signal increased over time. The greatest fold increases were observed at the lower and intermediate seeding densities, particularly 2.5 × 10^6^ cells/mL, which reached a 129.48-fold increase at T48, and 5 × 10^6^ cells/mL, which reached an 89.71-fold increase at T72 (Table 1).

#### 3.1.3. Relative Metabolic Activity, Relative Cell Density and Metabolic Efficiency Index

The percentage of the relative metabolic activity (RMA) at T0 decreased with decreasing initial seeding density, from 100.00% at 10 × 10^6^ cells mL^−1^ to 23.56% at 2.5 × 10^6^ cells mL^−1^. At T24, the highest RMA was observed at 5 × 10^6^ cells mL^−1^, whereas the highest relative cell density was observed at 10 × 10^6^ cells mL^−1^. At this time point, the metabolic efficiency index ranged from 0.60 at 7.5 × 10^6^ cells mL^−1^ to 2.04 at 5 × 10^6^ cells mL^−1^. At T48, RMA values ranged from 84.61% to 100.00% across the tested densities. The 10 × 10^6^ cells mL^−1^ condition showed proportionality between RMA and relative cell density, with a metabolic efficiency index of 1.00 and Δ of 0.00. In contrast, the 5 × 10^6^ and 2.5 × 10^6^ cells mL^−1^ conditions showed RMA values of 94.26% and 84.61%, respectively, despite relative cell densities of 10.37% and 8.22%. This corresponded to metabolic efficiency indices of 9.09 and 10.29. At T72, the highest RMA was observed at 5 × 10^6^ cells mL^−1^, while the highest relative cell density was observed at 7.5 × 10^6^ cells mL^−1^. The 5 × 10^6^ cells mL^−1^ condition showed an RMA of 100.00%, a relative cell density of 18.93%, a metabolic efficiency index of 5.28 and Δ of 0.81. The 10 × 10^6^ and 7.5 × 10^6^ cells mL^−1^ conditions showed metabolic efficiency indices close to 1 (Table 2).

### 3.2. Digestive Gland-Derived Cultures

#### 3.2.1. Time- and Density-Dependent Morphology of Digestive Gland-Derived Mussel Primary Cell Cultures

**Figure 7:**
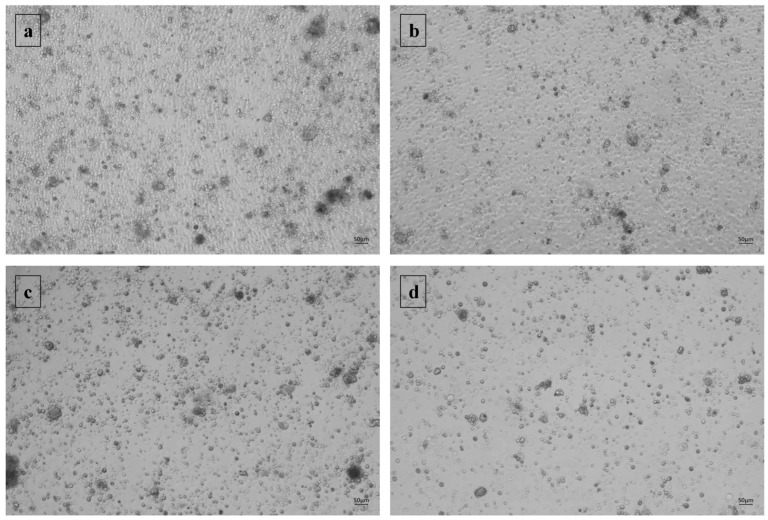
Representative light microscopy images (10×) of *M. galloprovincialis* digestive gland-derived cell suspensions immediately after seeding (T0) at different initial cell densities. Panels (**a**–**d**) correspond to 10 × 10^6^, 7.5 × 10^6^, 5 × 10^6^ and 2.5 × 10^6^ cells mL^−1^, respectively.

**Figure 8:**
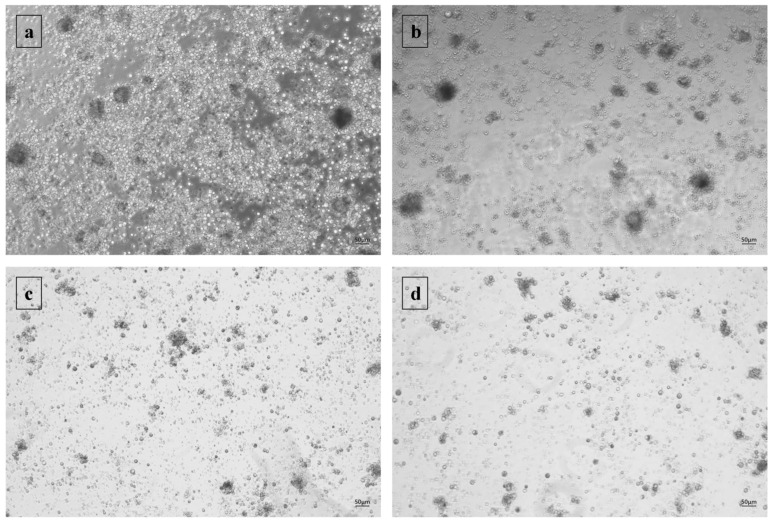
Representative light microscopy images (10×) of *M. galloprovincialis* digestive gland-derived cell suspensions immediately after 24 h of incubation (T24) at different initial cell densities. Panels (**a**–**d**) correspond to 10 × 10^6^, 7.5 × 10^6^, 5 × 10^6^ and 2.5 × 10^6^ cells mL^−1^, respectively.

**Figure 9:**
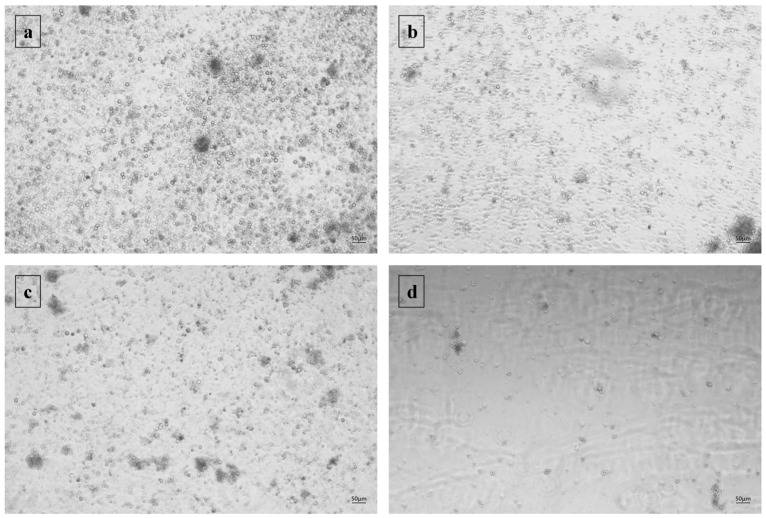
Representative light microscopy images (10×) of *M. galloprovincialis* digestive gland-derived cell suspensions immediately after 48 h of incubation (T48) at different initial cell densities. Panels (**a**–**d**) correspond to 10 × 10^6^, 7.5 × 10^6^, 5 × 10^6^ and 2.5 × 10^6^ cells mL^−1^, respectively.

**Figure 10:**
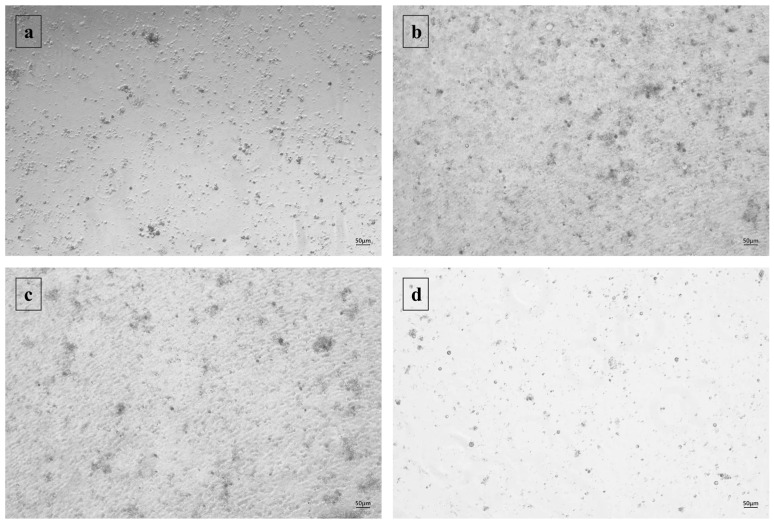
Representative light microscopy images (10×) of *M. galloprovincialis* digestive gland-derived cell suspensions immediately after 72 h of incubation (T72) at different initial cell densities. Panels (**a**–**d**) correspond to 10 × 10^6^, 7.5 × 10^6^, 5 × 10^6^ and 2.5 × 10^6^ cells mL^−1^, respectively.

**Figure 11:**
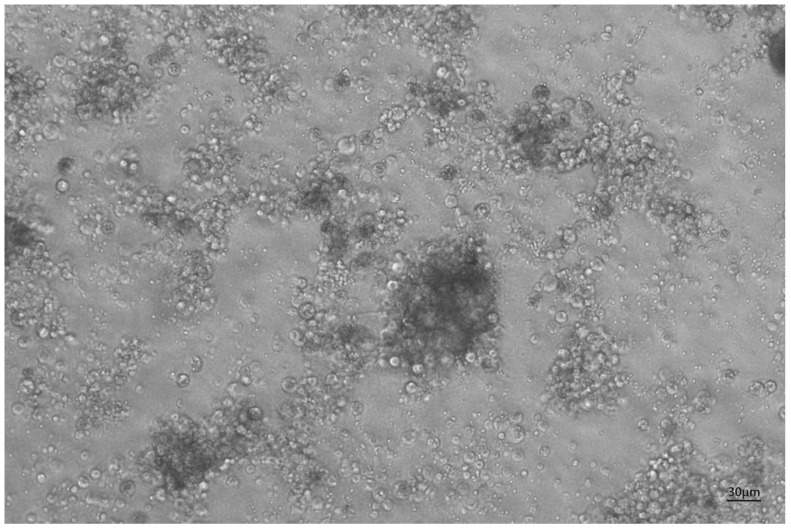
Detailed microscopic view of *M. galloprovincialis* digestive gland-derived cell suspensions at 20× magnification after 48 h of incubation at a cell density of 10 × 10^6^.

Figure 7, Figure 8, Figure 9 and Figure 10 illustrate the microscopic morphology of digestive gland-derived mussel primary cell cultures at T0, T24, T48 and T72, respectively. In each figure, panels a–d represent the initial seeding densities of 10 × 10^6^, 7.5 × 10^6^, 5 × 10^6^ and 2.5 × 10^6^ cells mL^−1^, respectively. Across all incubation times and seeding densities, digestive gland-derived cells displayed a predominantly rounded to spheroidal morphology. Individual cells were generally smaller than the 30 µm reference scale (Figure 11). Cell spatial organization was also influenced by the initial seeding density. At lower densities, cells were mainly observed as isolated or loosely distributed elements, with evident intercellular spaces and a predominantly non-confluent arrangement. At higher densities, cultures showed increased cellular proximity, reduced intercellular spacing and more frequent focal clustering or compact cellular aggregates. Compared with gill-derived cultures, the digestive gland cell suspension appeared more heterogeneous, with marked differences in optical density and aggregate compactness, consistent with the tissue-specific complexity of digestive gland preparations.

#### 3.2.2. Effects of Seeding Density on Fluorescence Intensity

**Figure 12:**
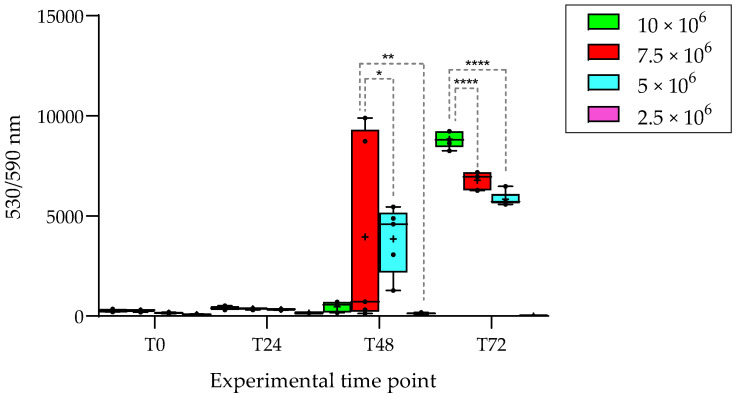
Time-dependent changes in fluorescence intensity in digestive gland-derived primary cultures. **** *p* < 0.0001; ** *p* < 0.01: * *p* < 0.05.

At T0, no significant differences were detected among the four initial seeding densities. A similar pattern was observed at T24, and comparisons between T0 and T24 were non-significant for all tested densities, indicating that metabolic activity remained relatively stable during the first 24 h of culture. At T48, the response became density-dependent. Cultures seeded at 7.5 × 10^6^ and 5 × 10^6^ cells mL^−1^ showed significantly higher fluorescence than the corresponding low-fluorescence conditions at T0 and T24, with *p* values ranging from 0.0018 to 0.0095. In contrast, fluorescence values at T48 for 10 × 10^6^ and 2.5 × 10^6^ cells mL^−1^ did not differ significantly from those recorded at T0 or T24. Within T48, fluorescence was significantly higher in the 7.5 × 10^6^ and 5 × 10^6^ cells mL^−1^ conditions than in both the 10 × 10^6^ and 2.5 × 10^6^ cells mL^−1^ conditions, indicating a stronger metabolic response at intermediate seeding densities (*p* = 0.0083–0.0122 and *p* = 0.0043–0.0064, respectively). At T72, fluorescence was significantly higher in cultures seeded at 10 × 10^6^, 7.5 × 10^6^ and 5 × 10^6^ cells mL^−1^ than in most T0 and T24 conditions, whereas the 2.5 × 10^6^ cells mL^−1^ condition remained comparable to the early time points. Within T72, fluorescence at 10 × 10^6^ cells mL^−1^ was significantly higher than at 5 × 10^6^ cells mL^−1^ (*p* = 0.0490), and all three higher-density conditions showed significantly greater fluorescence than the 2.5 × 10^6^ cells mL^−1^ (*p* < 0.0001) condition. Between T48 and T72, a significant increase was observed only at 10 × 10^6^ cells mL^−1^, whereas intermediate-density conditions remained statistically unchanged (Figure 12). Overall, these results indicate that metabolic activity was maintained during the first 24 h and subsequently became dependent on seeding density, with intermediate densities showing the strongest response at T48 and higher densities maintaining greater activity at T72.

The fluorescence signal also varied over time according to the initial seeding density. The highest fold increases were observed at 5 × 10^6^ cells mL^−1^, which reached a 37.36-fold increase at T72, at 10 × 10^6^ cells mL^−1^, which reached a 35.17-fold increase at T72, and at 7.5 × 10^6^ cells mL^−1^, which reached a 35.63-fold increase at T48. In contrast, the lowest seeding density, 2.5 × 10^6^ cells mL^−1^, showed a lower fold increase up to T48 and became non-determinable at T72, indicating the loss of a reliable metabolic signal under this condition (Table 3).

#### 3.2.3. Relative Metabolic Activity, Relative Cell Density and Metabolic Efficiency Index

The percentage of RMA at T0 ranged from 34.78% at 2.5 × 10^6^ cells mL^−1^ to 100.00% at 7.5 × 10^6^ cells mL^−1^. At T24, the 10 × 10^6^ cells mL^−1^ condition showed both RMA and relative cell density equal to 100.00%, with a metabolic efficiency index of 1.00 and Δ of 0.00. At the remaining densities, metabolic efficiency indices were above 1, ranging from 1.19 at 2.5 × 10^6^ cells mL^−1^ to 2.30 at 5 × 10^6^ cells mL^−1^. At T48, the 10 × 10^6^ cells mL^−1^ condition showed an RMA of 5.09%, while the relative cell density remained 100.00%, corresponding to a metabolic efficiency index of 0.05 and Δ of −0.95. At the same time point, 7.5 × 10^6^ cells mL^−1^ showed the highest RMA (100.00%), with a relative cell density of 54.35%, a metabolic efficiency index of 1.84 and Δ of 0.46. The 5 × 10^6^ cells mL^−1^ condition showed an RMA of 37.47%, relative cell density of 17.17% and metabolic efficiency index of 2.18. At T72, the 10 × 10^6^ cells mL^−1^ condition showed RMA and relative cell density equal to 100.00%, with a metabolic efficiency index of 1.00. The 5 × 10^6^ cells mL^−1^ condition showed an RMA of 67.85% and a relative cell density of 15.27%, corresponding to a metabolic efficiency index of 4.44 and Δ of 0.53. The 2.5 × 10^6^ cells mL^−1^ condition showed RMA and relative cell density equal to 0.00%; therefore, the metabolic efficiency index was not calculated (Table 4).

## 4. Discussion

The present study provides a methodological evaluation of short-term primary cultures derived from the gills and digestive gland of *M. galloprovincialis*, with specific emphasis on the role of initial seeding density in determining culture morphology, cell maintenance, and metabolic performance over time. This work is framed within the broader context of the early screening of xenobiotics, alongside the assessment of their environmental safety in non-target marine organisms.

Gill- and digestive gland-derived primary cell cultures from mussels therefore provide a suitable in vitro platform to capture early redox and metabolic perturbations, which are relevant both for the screening of antioxidant and anti-inflammatory activities and for the assessment of potential ecotoxicological effects in non-target marine organisms. By adapting the soft dissociation strategy previously described for *M. edulis* by Faucet et al. [29] and expanding the tested seeding-density range, this research addresses an important operational gap in mussel cell culture methodology. This gap is particularly relevant because standardized and reproducible bivalve in vitro models are needed not only for contaminant-response assessment, but also for the preliminary screening of xenobiotics, including marine drugs, natural products, and antifouling agents intended for aquatic applications [4]. For these bioactive compounds, ecologically relevant target or directly exposed species should be considered during early safety assessment. Although marine bivalve primary cultures have long been proposed as useful tools for ecotoxicological and mechanistic studies, their broader application remains constrained by incomplete standardization, tissue-specific variability, limited proliferative capacity, and uncertainty regarding optimal culture conditions [5,7,13,36]. The present findings support the view that mussel primary cultures should not be interpreted as long-term proliferative models, but rather as short-term, tissue-derived cellular systems suitable for assessing early functional responses under controlled in vitro conditions.

Microscopic observations showed that both gill- and digestive gland-derived cells maintained a predominantly rounded to spheroidal morphology throughout the observation period, with individual cells generally smaller than the 30 µm reference scale. This observation agrees with the methodological framework established by Faucet et al. [29], who reported the isolation and short-term maintenance of viable digestive gland cells from *M. edulis*, and with Gómez-Mendikute et al. [37], who characterized heterogeneous but highly viable gill cell suspensions from *M. galloprovincialis* for in vitro ecotoxicological applications. This morphological pattern is consistent with short-term molluscan primary cultures, which are generally characterized by heterogeneous tissue-derived cell populations, limited proliferative potential, and variable or delayed substrate attachment, rather than by the rapid establishment of stable epithelial monolayers during the initial culture period [29,36,37]. However, some tissue-specific differences were observed. Gill-derived cultures displayed a more homogeneous microscopic appearance, with smaller, regularly rounded cells and a more uniform spatial distribution. In contrast, digestive gland-derived cultures showed greater morphological heterogeneity, more variable optical density, cytoplasmic granularity, and a higher tendency to form compact aggregates. These differences are consistent with the biological organization of the two tissues. Mussel gills contain multiple epithelial and non-epithelial cell populations, including ciliated epithelial cells, non-ciliated cells, mucocytes and haemocytes, but previous work has shown that gill suspensions can be obtained with high initial viability and maintained as useful in vitro models for xenobiotic assessment [37]. Digestive gland preparations, by contrast, are expected to contain a more complex mixture of digestive cells, duct epithelial cells, haemocytes and lipid- or granule-rich cell types, reflecting the intracellular digestive, lysosomal and xenobiotic-processing functions of this organ [38].

Assessment of metabolic viability using the alamarBlue assay further indicated that short-term culture performance was modulated by both tissue origin and initial seeding density. Because resazurin reduction reflects the conversion of resazurin to resorufin by metabolically active cells, fluorescence intensity should be interpreted primarily as an indicator of cellular metabolic activity rather than as a direct measure of cell number [22,32,35]. This distinction is particularly relevant when assessing bioactive compounds with potential antioxidant or redox-modulating activity, as such compounds may differentially affect metabolic pathways across tissues. In the present study, fluorescence intensity did not always follow the same pattern, confirming that metabolic readouts alone may provide an incomplete representation of culture status. This is consistent with broader recommendations for metabolic viability assays, which emphasize that tetrazolium- and resazurin-based signals can be influenced by mitochondrial activity, redox state, assay duration, cell type and culture density, rather than by cell number alone [22,34,35]. The dissociation between metabolic activity and relative cell density was particularly evident in gill-derived cultures. At T48 and T72, intermediate- and low-density conditions displayed high metabolic activity despite comparatively lower relative cell density, resulting in elevated metabolic efficiency indices. This suggests that, under certain density conditions, the remaining viable cells may exhibit higher per-cell metabolic activity. Several mechanisms may contribute to this pattern. Lower density may reduce crowding, oxygen limitation and accumulation of metabolic by-products, thereby allowing surviving cells to maintain higher redox activity. Alternatively, the apparent increase in metabolic efficiency may reflect the selective survival of more metabolically active cell subpopulations after dissociation. However, because alamarBlue does not distinguish between increased per-cell activity, improved survival of specific cell types or true proliferation, these findings should not be interpreted as evidence of cell multiplication [16,20]. Proliferation-specific markers or metabolomic cell-cycle analysis would be required to determine whether any proliferative process contributes to the observed fluorescence increase.

In contrast, digestive gland-derived cultures displayed a more limited metabolic response, with a stronger dependence on initial seeding density. As observed for gills, fluorescence values remained relatively stable between T0 and T24, suggesting limited early changes in metabolic activity during the first day of culture. At T48, however, the response became strongly dependent on initial seeding density. Intermediate densities, particularly 7.5 × 10^6^ and 5 × 10^6^ cells mL^−1^, supported higher fluorescence responses, whereas the highest and lowest densities showed weaker performance. By T72, the 10 × 10^6^, 7.5 × 10^6^ and 5 × 10^6^ cells mL^−1^ conditions retained higher metabolic signals than the early time points, whereas the 2.5 × 10^6^ cells mL^−1^ condition showed poor stability. This pattern is consistent with the broader principle that seeding density can strongly influence metabolic assay performance, since cell density affects nutrient availability, waste accumulation, cell–cell interactions, and the linearity of viability assay readouts over time [15,20]. In addition, recent work on *M. edulis* primary cultures has shown that cells isolated from different tissues display distinct attachment, survival, and proliferative behavior in vitro [39], supporting a tissue-specific interpretation of culture performance.

To better interpret the relationship between metabolic output and cell abundance, the percentage of RMA, relative cell density and a metabolic efficiency index were calculated. These parameters were used as operational descriptors of metabolic activity relative to the maintained cell fraction, rather than as direct measures of cellular bioenergetic efficiency. This approach is consistent with recommendations for metabolic viability assays, which recognize that resazurin- and tetrazolium-based readouts are influenced not only by cell number, but also by cell type, metabolic state, culture density, incubation time and assay conditions [20,35,40]. Accordingly, normalizing metabolic activity to an independent estimate of cell abundance can help distinguish changes in total viable biomass from changes in activity per remaining cell fraction [41,42,43]. Although these parameters have not been specifically standardized for mussel primary cell cultures, comparable normalization strategies are widely used in cell viability and cytocompatibility studies. For example, Fan et al. [44] normalized MTT-derived metabolic activity to untreated controls and showed that initial cell density modulated treatment sensitivity in hepatocellular carcinoma cells, while Ryu et al. [45] expressed WST-1-derived metabolic activity relative to controls to evaluate proliferation-associated metabolic responses in human adipose-derived mesenchymal stem cells.

In both gill- and digestive gland-derived cultures, RMA followed the expected density-dependent pattern at T0 and T24; however, at T48 and T72, intermediate- and low-seeding densities displayed elevated RMA values despite comparatively reduced relative cell density, resulting in metabolic efficiency indices above 1. A notable observation in gill-derived cultures was the reduction in RMA at the highest seeding density (10 × 10^6^ cells mL^−1^) at T48, despite this condition retaining the highest relative cell density (100.00%), followed by full recovery at T72 (RMA = 100.00%). This pattern does not reflect cell loss followed by biological recovery, as cell counts remained stable between T48 and T72, confirming that the observed fluorescence fluctuation is not attributable to changes in cell viability per se. Two complementary mechanisms are proposed to account for this observation. First, because RMA represents a within-time-point relative measure, normalized to the highest absolute RFU value recorded among all cell-density conditions at each sampling time, the reduced RMA observed at T48 should be interpreted in relation to the internal reference for that specific time point. At T48, the 7.5 × 10^6^ cells mL^−1^ condition produced the highest absolute fluorescence signal and was therefore set as the reference value. Conversely, at T72, the 10 × 10^6^ cells mL^−1^ condition again exhibited the highest absolute fluorescence and consequently became the new reference condition. This shift in the time-point-specific reference explains the apparent recovery of RMA to 100% at T72. Second, a recognized technical limitation of resazurin-based metabolic assays is that, under conditions of high cell density and/or prolonged incubation, the assay may deviate from its linear detection range. Under such conditions, the available resazurin can become depleted, and resorufin, the main fluorescent reduction product of resazurin, may undergo further reduction to hydro-resorufin, a colorless and non-fluorescent compound. This secondary reduction step can result in an apparent decrease in fluorescence despite the presence of viable and metabolically active cells [46]. Therefore, the reduced fluorescence observed at the highest seeding density may reflect assay over-reduction rather than a true decline in metabolic activity.

A further interpretive caution applies to extreme metabolic efficiency index values, such as the index of 4.44 recorded at 5 × 10^6^ cells mL^−1^ at T72 in gill-derived cultures. Since this index is calculated as the ratio between RMA and relative cell density (both of which are within-time-point normalized values), disproportionately high results can arise when the denominator approaches very low values, amplifying the ratio independently of any true biological effect. Such values may nonetheless indicate that the surviving cell fraction maintains comparatively high metabolic output relative to its size, possibly reflecting the selective retention of more metabolically active subpopulations or the density-dependent upregulation of per-cell metabolic activity [47]. These indices should therefore be interpreted with caution and always considered in conjunction with absolute fluorescence values and cell density data.

In digestive gland-derived cultures, the response was more density-dependent: intermediate densities, especially 7.5 × 10^6^ and 5 × 10^6^ cells mL^−1^, provided the most favorable balance between RMA and relative cell density, whereas the highest density showed reduced efficiency at T48, and the lowest density lost a reliable metabolic signal by T72. Taken together, these findings indicate that intermediate seeding densities, particularly 5 × 10^6^–7.5 × 10^6^ cells mL^−1^, provide the most suitable compromise between cell maintenance and metabolic activity. Importantly, elevated metabolic efficiency should not be interpreted as evidence of proliferation, but rather as an indication of increased metabolic activity within the remaining viable cell fraction.

## 5. Conclusions

The contrasting behavior of gill- and digestive gland-derived cultures highlights the need for tissue-specific optimization of seeding density rather than the use of a single standard condition. Overall, intermediate densities, particularly 5 × 10^6^–7.5 × 10^6^ cells mL^−1^, appeared to provide the most suitable compromise between cell maintenance and metabolic activity. Digestive gland cultures were more density-dependent and less stable at the lowest density, whereas gill-derived cultures showed broader tolerance across the tested range and maintained strong metabolic responsiveness even at lower densities. The combined use of metabolic indices provided a more informative framework than fluorescence intensity or cell counts alone, allowing culture performance to be interpreted in relation to both metabolic output and relative cell abundance. This approach may improve the reproducibility and biological interpretation of short-term mussel primary culture assays. Moreover, by improving the reliability of short-term tissue-derived culture systems, this approach may contribute to the refinement of ecotoxicological testing strategies and to the reduction in extensive in vivo experimentation.

Nevertheless, some limitations should be acknowledged. In this context, gill- and digestive gland-derived primary cultures may represent useful complementary in vitro platforms for investigating early cellular responses to xenobiotics, particularly those targeting oxidative stress and immune/inflammation-like pathways. These marine bivalve primary cell cultures were evaluated only over a short time and alamarBlue fluorescence cannot distinguish between changes in cell number, redox activity, mitochondrial function, or cell-type composition. Future studies should therefore integrate metabolic assays with cell-type characterization, imaging-based quantification, lysosomal and oxidative stress endpoints, inflammation-related biomarkers, and molecular profiling to further refine mussel primary culture models for both ecotoxicological applications and the early screening of xenobiotics.

## Figures and Tables

**Table 1:** Temporal fold change in fluorescence signal in gill-derived primary cultures.

Initial Density (Cells/mL)	T0	T24 vs. T0	T48 vs. T0	T72 vs. T0
10 × 10^6^	1.00	1.03	36.05	33.65
7.5 × 10^6^	1.00	0.59	45.50	50.09
5 × 10^6^	1.00	2.43	73.88	89.71
2.5 × 10^6^	1.00	2.88	129.48	124.54

**Table 2:** Relative metabolic activity, cell density and metabolic efficiency in gill-derived primary cultures over time.

Time	Initial Density (Cells/mL)	% Relative Metabolic Activity (RMA)	% Relative Cell Density	Metabolic Efficiency Index
T0	10 × 10^6^	100.00	n.d.	n.d.
	7.5 × 10^6^	75.95	n.d.	n.d.
	5 × 10^6^	46.00	n.d.	n.d.
	2.5 × 10^6^	23.56	n.d.	n.d.
T24	10 × 10^6^	91.97	100.00	0.92
	7.5 × 10^6^	40.10	66.67	0.60
	5 × 10^6^	100.00	49.02	2.04
	2.5 × 10^6^	60.70	54.90	1.11
T48	10 × 10^6^	100.00	100.00	1.00
	7.5 × 10^6^	95.86	76.64	1.25
	5 × 10^6^	94.26	10.37	9.09
	2.5 × 10^6^	84.61	8.22	10.29
T72	10 × 10^6^	81.54	80.36	1.01
	7.5 × 10^6^	92.19	100.00	0.92
	5 × 10^6^	100.00	18.93	5.28
	2.5 × 10^6^	71.10	18.21	3.90

n.d.: not determined.

**Table 3:** Temporal fold change in fluorescence signal in digestive gland-derived primary cultures.

Initial Density (Cells/mL)	T0	T24 vs. T0	T48 vs. T0	T72 vs. T0
10 × 10^6^	1.00	1.70	1.88	35.17
7.5 × 10^6^	1.00	1.41	35.63	25.94
5 × 10^6^	1.00	2.08	21.65	37.36
2.5 × 10^6^	1.00	1.75	4.74	n.d.

**Table 4:** Relative metabolic activity, cell density and metabolic efficiency in gill-derived primary cultures over time.

Time	Initial Density (Cells/mL)	% Relative Metabolic Activity (RMA)	% Relative Cell Density	Metabolic Efficiency Index
T0	10 × 10^6^	96.50	n.d.	n.d.
	7.5 × 10^6^	100.00	n.d.	n.d.
	5 × 10^6^	61.65	n.d.	n.d.
	2.5 × 10^6^	34.78	n.d.	n.d.
T24	10 × 10^6^	100.00	100.00	1.00
	7.5 × 10^6^	85.99	66.87	1.29
	5 × 10^6^	78.37	34.06	2.30
	2.5 × 10^6^	37.23	31.16	1.19
T48	10 × 10^6^	5.09	100.00	0.05
	7.5 × 10^6^	100.00	54.35	1.84
	5 × 10^6^	37.47	17.17	2.18
	2.5 × 10^6^	4.63	11.09	0.42
T72	10 × 10^6^	100.00	100.00	1.00
	7.5 × 10^6^	76.43	65.45	1.17
	5 × 10^6^	67.85	15.27	4.44
	2.5 × 10^6^	0.00	0.00	n.d.

n.d.: not determined.

## Data Availability

The original contributions presented in this study are included in the article. Further inquiries can be directed to the corresponding author.

## Abbreviations

The following abbreviation is used in this manuscript:

RMA	Relative metabolic activity

## References

[1] Goldberg E.D., Bowen V.T., Farrington J.W., Harvey G.R., Martin J.H., Parker P.L., Risebrough R.W., Robertson W.D., Schneider E.D., Gamble E.J. (1978). The Mussel Watch. Environ. Conserv..

[2] Gosling E., Gosling E. (2003). Reproduction, Settlement and Recruitment. Bivalve Molluscs.

[3] Elskus A.A., LeBlanc L.A., Latimer J.S., Page D.S., Harding G.C.H., Wells P.G. (2020). Monitoring chemical contaminants in the Gulf of Maine, using sediments and mussels (*Mytilus edulis*): An evaluation. Mar. Pollut. Bull..

[4] Mayer A.M.S., Mayer V.A., Swanson-Mungerson M., Pierce M.L., Roberts C.M., Rodríguez A.D., Nakamura F., Taglialatela-Scafati O. (2026). Marine Pharmacology in 2022–2023: Marine Compounds with Antibacterial, Antidiabetic, Antifungal, Anti-Inflammatory, Antiprotozoal, Antituberculosis and Antiviral Activities, Affecting the Immune and Nervous Systems, and Other Miscellaneous Mechanisms of Action. Mar. Drugs.

[5] Potts R.W.A., Gutierrez A.P., Cortés-Araya Y., Houston R.D., Bean T.P. (2020). Developments in marine invertebrate primary culture reveal novel cell morphologies in the model bivalve Crassostrea gigas. PeerJ.

[6] Balakrishnan S., Singh I.S.B., Puthumana J. (2022). Status in molluscan cell line development in last one decade (2010–2020): Impediments and way forward. Cytotechnology.

[7] Domart-Coulon I., Blanchoud S. (2022). From primary cell and tissue cultures to aquatic invertebrate cell lines: An updated overview. Advances in Aquatic Invertebrate Stem Cell Research.

[8] Cima F., Varello R. (2023). Immunotoxic effects of exposure to the antifouling copper(I) biocide on target and nontarget bivalve species: A comparative in vitro study between *Mytilus galloprovincialis* and *Ruditapes philippinarum*. Front. Physiol..

[9] Impellitteri F., Curpăn A.S., Plăvan G., Ciobica A., Faggio C. (2022). Hemocytes: A Useful Tool for Assessing the Toxicity of Microplastics, Heavy Metals, and Pesticides on Aquatic Invertebrates. Int. J. Environ. Res. Public Health.

[10] Carroll A.R., Copp B.R., Grkovic T., Keyzers R.A., Prinsep M.R. (2025). Marine natural products. Nat. Prod. Rep..

[11] Fonseca S., Amaral M.N., Reis C.P., Custódio L. (2023). Marine Natural Products as Innovative Cosmetic Ingredients. Mar. Drugs.

[12] Blanco J., Martín H., Mariño C., Rossignoli A.E. (2019). Simple diffusion as the mechanism of okadaic acid uptake by the mussel digestive gland. Toxins.

[13] Rinkevich B., Pomponi S.A. (2025). Advancing marine invertebrate cell line research: Four key knowledge gaps. Vitr. Cell. Dev. Biol.-Anim..

[14] Freshney R.I., Freshney R.I. (2012). Biology of Cultured Cells. Culture of Animal Cells.

[15] Asiri A., Tasleem M., Al Said M., Asiri A., Al Qarni A.A., Bakillah A. (2025). Optimizing Cell Density and Unveiling Cytotoxic Profiles of DMSO and Ethanol in Six Cancer Cell Lines: Experimental and In Silico Insights. Methods Protoc..

[16] Clegg J., Curvello R., Gabrielyan A., Croagh D., Hauser S., Loessner D. (2025). Tailoring metabolic activity assays for tumour-engineered 3D models. Biomater. Adv..

[17] Nagai H., Miwa A., Yoneda K., Fujisawa K., Takami T. (2023). Optimizing the Seeding Density of Human Mononuclear Cells to Improve the Purity of Highly Proliferative Mesenchymal Stem Cells. Bioengineering.

[18] Ligasová A., Koberna K. (2019). Quantification of fixed adherent cells using a strong enhancer of the fluorescence of DNA dyes. Sci. Rep..

[19] Ziemba B. (2025). Advances in Cytotoxicity Testing: From In Vitro Assays to In Silico Models. Int. J. Mol. Sci..

[20] Petiti J., Revel L., Divieto C. (2024). Standard Operating Procedure to Optimize Resazurin-Based Viability Assays. Biosensors.

[21] van Tonder A., Joubert A.M., Cromarty A.D. (2015). Limitations of the 3-(4,5-dimethylthiazol-2-yl)-2,5-diphenyl-2H-tetrazolium bromide (MTT) assay when compared to three commonly used cell enumeration assays. BMC Res. Notes.

[22] O’Brien J., Wilson I., Orton T., Pognan F. (2000). Investigation of the Alamar Blue (resazurin) fluorescent dye for the assessment of mammalian cell cytotoxicity. Eur. J. Biochem..

[23] Regoli F., Giuliani M.E. (2014). Oxidative pathways of chemical toxicity and oxidative stress biomarkers in marine organisms. Mar. Environ. Res..

[24] Multisanti C.R., Zicarelli G., Caferro A., Filice M., Faggio C., Vazzana I., Blahova J., Lakdawala P., Cerra M.C., Imbrogno S. (2024). From personal care to coastal concerns: Investigating polyethylene glycol impact on mussel’s antioxidant, physiological, and cellular responses. Antioxidants.

[25] Liberatori G., Grassi G., Guidi P., Bernardeschi M., Fiorati A., Scarcelli V., Genovese M., Faleri C., Protano G., Frenzilli G. (2020). Effect-Based Approach to Assess Nanostructured Cellulose Sponge Removal Efficacy of Zinc Ions from Seawater to Prevent Ecological Risks. Nanomaterials.

[26] Galloway T.S., Depledge M.H. (2001). Immunotoxicity in invertebrates: Measurement and ecotoxicological relevance. Ecotoxicology.

[27] Canesi L., Gallo G., Gavioli M., Pruzzo C. (2002). Bacteria–hemocyte interactions and phagocytosis in marine bivalves. Microsc. Res. Tech..

[28] Fernández B., Campillo J.A., Martínez-Gómez C., Benedicto J. (2012). Assessment of the mechanisms of detoxification of chemical compounds and antioxidant enzymes in the digestive gland of mussels, *Mytilus galloprovincialis*, from Mediterranean coastal sites. Chemosphere.

[29] Faucet J., Maurice M., Gagnaire B., Renault T., Burgeot T. (2003). Isolation and primary culture of gill and digestive gland cells from the common mussel *Mytilus edulis*. Methods Cell Sci..

[30] Balakrishnan S., Sajeevan A.K.M., Parvathi S.C., Bright Singh I.S., Puthumana J. (2024). An optimized protocol for routine development of cell culture from adult oyster, *Crassostrea madrasensis*. Cell Biol. Int..

[31] Udayan G., Giordano M.E., Pagliara P., Lionetto M.G. (2023). Motility of *Mytilus galloprovincialis* hemocytes: Sensitivity to paracetamol in vitro exposure. Aquat. Toxicol..

[32] Rampersad S.N. (2012). Multiple applications of Alamar Blue as an indicator of metabolic function and cellular health in cell viability bioassays. Sensors.

[33] Mosmann T. (1983). Rapid colorimetric assay for cellular growth and survival: Application to proliferation and cytotoxicity assays. J. Immunol. Methods.

[34] Stockert J.C., Blázquez-Castro A., Cañete M., Horobin R.W., Villanueva Á. (2012). MTT assay for cell viability: Intracellular localization of the formazan product is in lipid droplets. Acta Histochem..

[35] Riss T.L., Moravec R.A., Niles A.L., Duellman S., Benink H.A., Worzella T.J., Minor L., Markossian S., Grossman A., Baskir H., Arkin M., Auld D., Austin C., Baell J., Brimacombe K., Chung T.D.Y., Coussens N.P. (2016). Cell viability assays. Cell Viability Assays. Assay Guidance Manual [Internet].

[36] Yoshino T.P., Bickham U., Bayne C.J. (2013). Molluscan cells in culture: Primary cell cultures and cell lines. Can. J. Zool..

[37] Gómez-Mendikute A., Elizondo M., Venier P., Cajaraville M.P. (2005). Characterization of mussel gill cells in vivo and in vitro. Cell Tissue Res..

[38] Birmelin C., Pipe R.K., Goldfarb P.S., Livingstone D.R. (1999). Primary cell-culture of the digestive gland of the marine mussel *Mytilus edulis*: A time-course study of antioxidant-and biotransformation-enzyme activity and ultrastructural changes. Mar. Biol..

[39] Hosseini Khorami H., Breton S., Angers A. (2024). In vitro proliferation of *Mytilus edulis* male germ cell progenitors. PLoS ONE.

[40] Uzarski J.S., DiVito M.D., Wertheim J.A., Miller W.M. (2017). Essential design considerations for the resazurin reduction assay to noninvasively quantify cell expansion within perfused extracellular matrix scaffolds. Biomaterials.

[41] Pinho S.A., Oliveira P.J., Cunha-Oliveira T. (2025). Heterogeneous redox responses in NHDF cells primed to enhance mitochondrial bioenergetics. Biochim. Biophys. Acta (BBA)-Mol. Basis Dis..

[42] Shama K.A., Greenberg Z.F., Tammame C., He M., Taylor B.L. (2024). Diseased Tendon Models Demonstrate Influence of Extracellular Matrix Alterations on Extracellular Vesicle Profile. Bioengineering.

[43] Ruhl T., Benic S., Plum M., Kim B.S., Beier J.P., Schaefer B. (2025). ∆ 9-Tetrahydrocannabinol Increases Growth Factor Release by Cultured Adipose Stem Cells and Adipose Tissue in vivo. Tissue Eng. Regen. Med..

[44] Fan H.-L., Liu S.-T., Chang Y.-L., Chiu Y.-L., Huang S.-M., Chen T.-W. (2022). In vitro cell density determines the sensitivity of hepatocarcinoma cells to ascorbate. Front. Oncol..

[45] Ryu C., Lee M., Lee J.Y. (2023). Mild heat treatment in vitro potentiates human adipose stem cells: Delayed aging and improved quality for long term culture. Biomater. Res..

[46] Gong X., Liang Z., Yang Y., Liu H., Ji J., Fan Y. (2020). A resazurin-based, nondestructive assay for monitoring cell proliferation during a scaffold-based 3D culture process. Regen. Biomater..

[47] Estevens R., Heinrichs A.L., Ghedini G. (2026). Drivers of metabolic density-dependence: How resource availability and conspecific cues affect phytoplankton metabolism. Oikos.

